# The Prevent programme: an ethical dilemma for teachers as well as psychiatrists

**DOI:** 10.1192/pb.bp.116.053611

**Published:** 2016-04

**Authors:** Stephanie Reed

**Affiliations:** 1 State secondary school, London, UK

## Abstract

The UK government's Prevent programme affects professionals and the people who rely on their services across the public sector, particularly now that workers are legally bound to report their concerns about individuals they believe to be at risk of radicalisation. This article discusses the risks that the strategy presents to the work of teachers and the bonds of trusts between staff and students.

Many teachers have raised concerns about Prevent,^1^ the UK government's anti-radicalisation programme, which requires public sector workers to report (so-called) ‘at-risk’ individuals. Criticisms centre around the fallacy that underpins the programme: that certain individuals must have specific, identifiable ‘psychological vulnerabilities’ that make them more likely to engage in terrorism. Meanwhile, most research (including leaked guidance provided to the Cabinet) states that radicalisation is not ‘a linear “conveyor belt” moving from grievance, through radicalisation, to violence’.^2^ Teachers also worry about the lack of protection for the staff now legally obliged to report their concerns, and the dangers of inviting teachers to profile students based on their race and religion. Serious, almost inevitable, abuses have been reported. A student was referred to Channel (the multi-agency panel, part of the Prevent programme, which focuses on providing support at an early stage to people who are identified as being vulnerable to being drawn into terrorism; the panel must include a police representative) for wearing a ‘free Palestine’ badge; another for quoting ‘the history of the Caliphate’ in his homework; a third interviewed about his involvement with IS after saying the word ‘eco-terrorism’ when discussing environmental activism.^3^ More worrying, however, is the day-to-day effect Prevent has on all Muslim students.

It is axiomatic that education means more than memorising facts and regurgitating them in an exam; it includes developing skills and abilities and broadening intellectual and emotional horizons. Perhaps most important is what Bernard Crick^4^ called ‘political literacy’: the ability to think critically about, question, and engage with the political process. Educational researcher Dr Aminul Hoque recently listed behaviour which might exemplify this, including ‘speaking out against social injustice’, ‘challenging the status quo’ and ‘questioning those in power to be held accountable for their actions’.^5^ This is disturbingly similar to some of the ‘warning signs’ that a young person is vulnerable to radicalisation. For example, a leaflet recently distributed to parents in north London described ‘showing a mistrust of mainstream media reports’ and ‘appearing angry about government policies’ as traits ‘specific to radicalisation’.^6^ In essence, this is what teachers are told to watch out for, not as signs that we have done our job properly, but as signs that our students are potential dangers to society. Teachers have a duty to create a free and safe space for the type of discussions that help our students become intelligent, active members of society. Prevent hinders that.

The effects will be felt most in subjects that necessitate discussion of controversial issues, such as politics. However, curriculum content aside, most of my students are politically engaged young Londoners: they come in each morning bursting to discuss the latest news and, like many of us, have grievances to air. Something will be lost from their education if they no longer feel safe doing this with, or even in front of, their teachers. Research shows that students perform better academically if importance is attributed to issues that matter to them personally;^5^ pretending that issues such as racism, community divides and radicalisation are somehow off-limits for discussion is probably the most dangerous thing we could do.

Forcing a consensus papers over the problem rather than solving it. We should be encouraging students to speak openly and showing them that it is not a crime to disagree with someone or have ‘difficult’ opinions, even if that means they say things we dislike. This approach would allow children to open up to their teachers, rather than keeping opinions a secret; only then can we try to talk them down, address their concerns or try to reshape their outlook. This would require a bond of trust between student and teacher. If students feel they cannot trust teachers with their opinions, or worries, they will find other outlets to explore their ideas, and organisations such as IS are adept at fostering these spaces in the dark shadows of the internet. But how can our students trust us, if we are legally obliged to report anything other than government-approved opinions?

In my school, where around 85% of students are Muslim, Prevent has become the subject of jokes; last term a group of girls threatened to report me for trying to indoctrinate them into an ancient cult (I teach Latin). For staff, meanwhile, Prevent effectively tells us that, on discovering that a student suffers from depression, isolation or a difficult home life, our first reaction should be to start a process that would end with a police investigation. For teachers, who have their students' welfare at heart, this reaction is inconceivable; referring a teenager already suffering from social exclusion to the police seems counter-productive and designed to create the very issue it seeks to prevent. IS for one capitalises on feelings of isolation, drawing its recruits closer the more they feel ostracised from their own society. By encouraging teachers to treat Muslim students and their problems differently, Prevent could exacerbate one of the central issues that drive teenagers to extremism.

Deeyah Khan, maker of the documentary *Jihad: A British Story*, revealed that IS spends hundreds of hours recruiting each member via Skype, an online communicator: ‘IS takes the yearning, the sadness, the anger, preys on that and draws people into becoming cannon fodder […] It starts out as a human need that is not being met, and with love and loyalty between the recruiter and the follower’.^7^ Who would be better placed to compete with this level of care and attention than teachers? We spend many hours a day with our students, know them well and genuinely care about them; most of us would hope that students have at least one person at school whom they trust, with whom they would feel able to share anything – something they might not get anywhere else. Prevent risks fostering mistrust between students and teachers, and destroying that invaluable bond.

The Prevent strategy is right in its assumption that education is key in countering radicalisation, but its approach is wrong. Rather than turning us into spies, shutting down spaces for discussion and forcing students to censor themselves, we should be allowed to do what we do best: encourage free speech, discussion and thought, develop the critical skills that will allow young people to see through extremist rhetoric, and show students we are there to help them confront the issues that affect them.
